# Difficult Spinal-Arachnoid Puncture (DSP) Score: Development and Performance Analysis

**DOI:** 10.7759/cureus.33760

**Published:** 2023-01-14

**Authors:** Habib Md R Karim

**Affiliations:** 1 Anesthesiology, Critical Care, and Pain Medicine, All India Institute of Medical Sciences, Raipur, IND

**Keywords:** lumbar puncture (lp), spinal puncture, scores, interpretable machine learning, regional anesthesiology, orthopedic anesthesia, spinal deformiites

## Abstract

Background: Difficult and traumatic neuraxial blocks and procedures are not uncommon. Although score-based prediction has been attempted, the practical application of those has remained limited for various reasons. The aim of this study was to develop a clinical scoring system from the strong predictors of failed spinal-arachnoid puncture procedures assessed previously using artificial neural network (ANN) analysis and analyze the score’s performance on the index cohort.

Methods: The present study is based on the ANN model analyzing 300 spinal-arachnoid punctures (index cohort) performed in an academic institute in India. For the development of the score, i.e., Difficult Spinal-Arachnoid Puncture (DSP) Score, the coefficient estimates of the input variables, which showed a Pr(>|z|) value of <0.01, were considered. The resultant DSP Score was then applied to the index cohort for receiver operating characteristic (ROC) analysis, Youden’s J point determination for best sensitivity and specificity, and diagnostic statistical analysis for the cut-off value for predicting the difficulty.

Results: A DSP Score incorporating spine grades, performers’ experience, and positioning difficulty was developed; the minimum and maximum scores were 0 and 7, respectively. The area under the ROC curve for the DSP Score was 0.858 (95% confidence interval 0.811-0.905), Youden’s J point for cut-off was at 2, which showed a specificity and sensitivity of 98.15% and 56.5%, respectively.

Conclusion: The ANN model-based DSP Score developed for predicting the difficult spinal-arachnoid puncture procedure showed an excellent area under the ROC curve. At the cut-off value 2, the score had a sensitivity plus specificity of approximately 155%, indicating that the tool can be useful as a diagnostic (predictive) tool in clinical practice.

## Introduction

Spinal-arachnoid puncture procedures are commonly performed in healthcare practice for spinal anesthesia, diagnostic lumber puncture, and lumbar drainage. While the procedure is straightforward and seemingly effortless, studies have shown that first-pass success and the atraumatic puncture rates are relatively low [1-3]. Many studies have analyzed spinal anesthesia procedures and tried to find the factors that lead to difficult procedures requiring multiple passes and puncture attempts. Such multiple attempts can even lead to trauma and procedural failure. The difficult procedure might be associated with the technical aspect of the performer, anatomy of the patients' back , or other procedural aspects such as positioning difficulty. As difficult procedures bear importance for patient discomfort and satisfaction, it becomes prudent for the anesthesiologists to discuss these aspects during informed consent. Therefore, predicting difficult spinal-arachnoid procedures is crucial for providing quality perioperative care.

Over time, univariate analysis, multivariate analysis, logistic regressions, and recently, artificial neural network analysis have been employed to determine the factors attributable to the increased need for needle passes and punctures [1-3]. Cohorts from an extensive national database comprising 73,579 patients are also used [4]. The results, however, show remarkable variability. Furthermore, the development of a score from multiple predictors is still being determined. Atallah et al. developed one score in 2014 where the authors included clinical and radiological parameters [1]. The study by Khoshrang et al. also tried to find a score and cut-off value for predicting difficult procedures [5]. However, it was not apparent how much weightage and score were provided to the different parameters. Furthermore, this study also included radiographic features. On the other hand, Del Buono et al. developed one neuraxial block assessment score published in 2021, limiting the parameters to only clinical assessment [6]. They also included the past history of such difficulty. Radiological features and past history might have advantages, but not all patients need radiological spinal evaluation before spinal anesthesia, nor do most patients undergo repeated spinal anesthesia. Furthermore, beyond patient characteristics, performers' experience might impact the procedures [7]. The recently conducted deep machine learning-based study, which employed artificial neural network (ANN) analysis to find the significant predictors, also found that the performer's experience is a crucial determinant [3]. Therefore, we aimed to develop a score for predicting difficult spinal-arachnoid puncture procedures from the significant factors of this ANN-based study and analyze the diagnostic accuracy of the developed score on the index cohort, i.e., the cohort on which the ANN was applied.

## Materials and methods

The present study is the secondary analysis of a research proposal approved by our Institute Research Cell (project code no. AIIMS-RPR/IRC/IM/NF/2022/433) and Institute Ethics Committee, All India Institute of Medical Sciences, Raipur (No.2484/IEC-AIIMSRPR/2022) with consent waiver. The published ANN article describes the study settings, participants, definitions, data collection, and statistical analysis [3].

As the study uses the results of the ANN study as mentioned, no new sample size calculation was performed, and the diagnostic testing of the score developed was also performed on the index cohort of 300 procedures of the original study. However, the predictors are used to develop difficult spinal puncture procedures, not for failure. It was because a robust, universally accepted definition for failed spinal-arachnoid puncture procedure was not found in the literature. Many anesthesiologists and researchers traditionally reserve and use the term ‘failed spinal’ to describe the spinal-arachnoid block, which did not result in surgical anesthesia, required supplementation with sedation and analgesia, or even needed to be converted to general anesthesia [7,8]. The study by Atallah et al., Khoshrang et al., and Del Buono et al. used the term difficult spinal procedure concerning the need for multiple needle punctures and passes [1,5,6]. However, there is variation in the number of passes and punctures taken to define the difficult procedure. In the present study, the spinal-arachnoid puncture procedure was categorized as difficult if a performer required more than three punctures, with three punctures but more than six passes, or if the performer handed over the procedure to another performer and considered it as difficult after the second puncture. The present definition was adapted from the study by Atallah et al. who allowed residents to proceed with a maximum of two punctures followed by a senior taking over the procedure, and Khoshrang et al. who categorized the puncture as difficult when the performer attempted a third intervertebral space puncture [1,5].

Significant predictor selection and weightage

In the first step, the predictors that showed a significant Pr(>|z|) value, i.e., <0.01, were selected to include in the Difficult Spinal-Arachnoid Puncture (DSP) Score. In the second step, ANN estimate values of those significant factors were chosen as the weightage of the parameters. We have chosen the value to the nearest integer of 1. In the third step, positive and negative signs of the coefficient values were considered; a negative sign indicated that as the grading of the predictive parameter would increase, it would receive a higher score or vice versa.

Statistical analysis

The incidence of difficult spinal-arachnoid procedures was calculated using the data for the number of passes and punctures. Furthermore, the DSP Score was calculated for each procedure. Statistical analysis for the DSP Score to generate receiver operating characteristic (ROC) curves, area under the ROC curves (AUC), was performed online using Epitools epidemiological calculators [9]. Furthermore, Youden's J point analysis was also done to find the cut-off value of the DSP Score at which it had the optimal sensitivity and specificity. In addition, statistical calculator MedCalc was used for diagnostic statistics, including accuracy specific to difficult procedure prevalence in our study [10].

## Results

Among the 300 procedures, 54 (18%) fulfilled the difficult procedure criteria. The cohort characteristics and other procedure-related parameters were compared for difficult and non-difficult (easy) procedures and are presented in Table 1; weight and body mass index (BMI) was higher for difficult cases in univariate analysis.

**Table 1:** Comparison of different input parameters among the difficult and easy spinal-arachnoid puncture groups ASA-PS, American Society of Anesthesiologists Physical Status; SD, standard deviation; TLS, thoraco-lumbo-sacral N indicates the total number of the respective groups. ^a^Chi-square test for independence.

Parameters	Group difficult (N=54)	Group easy (N=246)	P-value
Age (years), mean ± SD	46.77 ± 16.06	45.03 ± 16.7	0.487
Sex, male/female, n (%)	40 (74.07)/14 (25.92)	187 (76.01)/59 (23.98)	0.729
Height (cm), mean ± SD	161.1 ± 10.38	161.9 ± 9.00	0.573
Weight (kg), mean ± SD	70.14 ± 18.02	60.56 ± 13.3	0.0001
Body mass index, mean ± SD	26.90 ± 5.84	23.07 ± 4.72	0.0001
ASA-PS class I, n (%)	12 (22.22)	64 (26.01)	0.054^a^
ASA-PS class II, n (%)	26 (48.14)	143 (58.13)	
ASA-PS class III, n (%)	13 (24.07)	36 (14.63)	
ASA-PS class IV, n (%)	3 (5.55)	3 (1.21)	
Elective/Emergency, n (%)	52 (96.29)/2 (0.03)	239 (97.1)/7 (2.84)	0.667
Spine grade 1, n (%)	1 (1.85)	94 (38.2)	0.0001^a^
Spine grade 2, n (%)	23 (42.5)	125 (50.8)	
Spine grade 3, n (%)	26 (48.1)	26 (10.5)	
Spine grade 4, n (%)	4 (7.40)	1 (0.40)	
Experience, 12/>12 months, n (%)	43 (79.62)/11 (20.38)	153 (62.19)/93 (37.21)	0.017
TLS couture - convex, n (%)	15 (27.77)	137 (55.69)	0.0001^a^
TLS couture - straight, n (%)	31 (57.40)	102 (41.46)	
TLS couture - concave, n (%)	8 (14.81)	7 (2.84)	
Positioning difficulty, n (%)	4 (7.40)	8 (3.25)	0.239
Spine deformity, n (%)	1 (1.85)	6 (2.43)	1.000
Patient position - sitting/lateral, n (%)	54 (100)/0	240 (97.5)/6 (2.43)	0.5107^a^

The ANN model showed a significant Pr(>|z|) value of <0.01 only for spinal grades, positioning difficulty, and performers' experience, with an estimate of -2.715, -2.98, and 1.045, respectively [3]. Accordingly, the DSP Score provided a maximum weightage of 3 for spinal grades and positioning difficulty and 1 for the performer's experience. The resulting DSP Score is shown in Table 2, which had a minimum possible aggregate value of 0 and a maximum of 7.

**Table 2:** Descriptions and scores assigned for individual parameters in the Difficult Spinal-Arachnoid Puncture Score ANN, artificial neural network

Parameters	ANN estimate	Descriptions	Score assigned
Spine grade	-2.715	Grade 1, spinous processes and intervertebral space can be guessed by inspection	0
		Grade 2, spinous processes and intervertebral space can be felt by superficial palpation	1
		Grade 3, spinous processes and intervertebral space can be felt by deep (with pressure) palpation	2
		Grade 4, spinous processes and intervertebral space cannot be felt even by deep palpation	3
Experience	1.045	Up to one year	1
		More than one year	0
Positioning difficulty	-2.98	Absent	0
		Present	3

**Figure 1:**
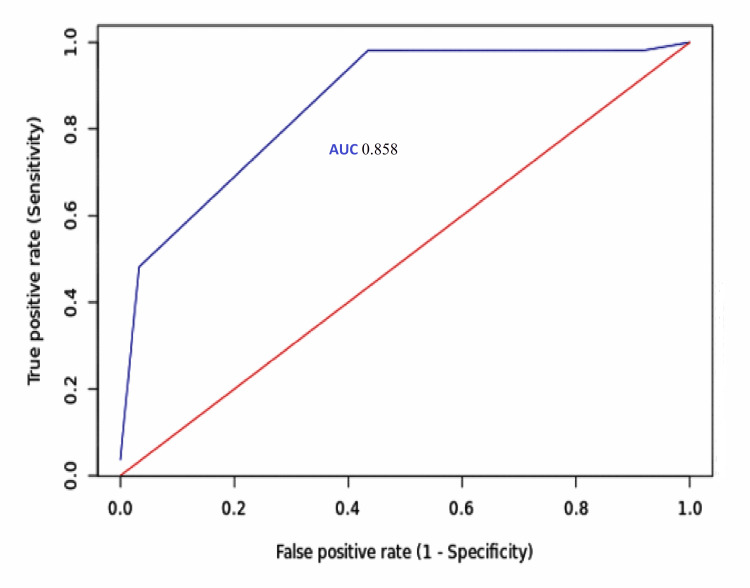
AUC for the Difficult Spinal-Arachnoid Puncture Score for predicting difficult spinal-arachnoid puncture procedures AUC, area under the receiver operating characteristic curve

On applying the DSP Score on the index cohort, the ROC curve showed very good AUC (0.858, 95% confidence interval 0.811-0.905) (Figure 1).

Youden’s J point was found at the cut-off value of 2 for the DSP Score. The test efficiency results are presented in Table 3.

**Table 3:** Youden’s J point analysis for efficiencies at different P-values and respective sensitivity and specificity of the Difficult Spinal-Arachnoid Puncture Score Eff., efficiency

Parameters	Cut-off point	Sensitivity	Specificity
Youden's J	2	0.981	0.565
Eff. at P = 0.01	4	0.037	1
Eff. at P = 0.05	4	0.037	1
Eff. at P = 0.1	3	0.481	0.967
Eff. at P = 0.2	3	0.481	0.967
Eff. at P = 0.3	3	0.481	0.967
Eff. at P = 0.5	2	0.981	0.565

The diagnostic accuracy testing for a DSP Score 2 for predicting difficult procedures showed excellent sensitivity, and negative predictive value, with good sensitivity and accuracy (Table 4).

**Table 4:** Results of the diagnostic testing statistics of the cut-off point DSP Score for predicting difficulty DSP Score, Difficult Spinal-Arachnoid Puncture Score; PLR, positive likelihood ratio; NLR, negative likelihood ratio; PPV, positive predictive value; NPV, negative predictive value; CI, confidence interval ^a^Values are specific for 18% prevalence.

Category	Difficult	Easy
DSP Score >2	53 (17.67%)	107 (35.67%)
DSP Score <2	1 (0.33%)	139 (46.33%)
Incidence	18.0%	82.0%
Statistics	Value	95% CI
Sensitivity	98.15%	90.11-99.95%
Specificity	56.50%	50.06-62.79%
PLR	2.26	1.95-2.61
NLR	0.03	0.00-0.23
Prevalence^a^	18.00%	
PPV^a^	33.12%	29.95-36.46%
NPV^a^	99.29%	95.21-99.90%
Accuracy^a^	64.00%	58.28-69.44%

## Discussion

Planning and preparation for any clinical procedure are of utmost importance for a better result. Studies have shown that difficult spinal anesthesia procedures requiring multiple needle pricks and redirection leading to repeated bony encounters might contribute to back pain [11]. Such repeated pricks and pain also lead to patient dissatisfaction and even have been attributed to spinal anesthesia refusal [12,13]. On the other hand, personalized risk communication and counseling have reduced patient dissatisfaction [14]. The development of the DSP Score was done keeping in mind identifying the patients who might have difficult spinal-arachnoid puncture procedures. It will allow the healthcare provider to do individualized preoperative counseling and proper planning of the procedure.

Compared to the other three scores, the current score has a lower number of variables [1,5,6]. However, the performance analysis showed that it is equally efficient. The score developed by Atallah et al. has five (age, BMI, bony spinal landmark, bony deformity, and radiological features), the one by Khoshrang et al. has four (BMI, radiological features, spinal bony deformity, and difficult to find spinous process), and the score by Del Buono et al. has four (previous history of difficult spinal anesthesia, spinous process visibility, spinous process palpability, and spinal deformity) variables [1,5,6]. On the other hand, the present DSP Score has three variables; one is related to the patient spinal space grade, one is related to the performer experience, and the last one is related to the patient and procedural environment, i.e., position difficulty. Age was considered by Atallah et al., but none of the other scores. The base ANN study on which the current DSP Score was developed also failed to find age as a significant predictor [3].

Furthermore, our study did not find BMI as a variable like Del Buono et al. [6]. Atallah et al. and Khoshrang et al. considered the performer's experience while designing the study but failed to find it as a significant variable [1,5]. The reason might be the variation in the experience accounted for by the studies where Atallah et al. took 6-12 months of training as the comparator. Furthermore, training procedure, duration, rotation, case exposure, and the timing of direct contact with the patient are different in different countries, especially in India, where the population is high compared to the relatively lacking healthcare facilities and a low number of anaesthesiologists [15].

Despite not considering the radiological features and past history of difficulty, the AUC and diagnostic accuracy of the DSP Score developed in the present study were very comparable, even better than others. The AUC of the Atallah et al. score was up to 0.78, and the Khoshrang et al. study using the Hosmer-Lemeshow goodness-of-fit test found a value of 0.78 [1,5]. On the contrary, the current score had an AUC of 0.858. At the cut-off value, the neuraxial block assessment score of Khoshrang et al. had a sensitivity of 86%, and the present DSP Score had 98% sensitivity. Nevertheless, the DSP Score developed fulfills the criteria to be a helpful screening tool that requires an AUC >0.7 or a sensitivity + specificity of >150% [16,17].

The present study has a few limitations as well. The score is developed and tested on the index cohort, which indicates that the power of the study might be inadequate for individual variables as mentioned in the base ANN study [3]. Furthermore, the index cohort had fewer obese and elderly patients; even the gender distribution was skewed toward males. Furthermore, pregnant patients have altered physiology and anatomy of the spine due to gravid uterus, hormonal changes, and weight gain, and even positioning for spinal anesthesia in such patients is frequently sub-optimal. Therefore, the current score cannot be generalized and will require further studies for diverse populations and demographics.

## Conclusions

The DSP Score developed from the statistically significant predictors from the ANN model could identify the difficult spinal-arachnoid puncture procedures requiring more than three punctures or with three punctures but more than six passes accurately. The score showed excellent sensitivity (i.e., 98%) at a value of 2 and specificity (i.e., 96.7%) at 3. The DSP Score also showed an excellent AUC (0.858) for the index cohort. Therefore, the score warrants validation, preferably from a multi-center study with a larger prospective design.
